# IR-780 improves urination function and complications in rats with partial bladder outlet obstruction by protecting bladder smooth muscle cell mitochondria from oxidative stress

**DOI:** 10.3389/fphar.2026.1778496

**Published:** 2026-02-27

**Authors:** Feng Pi, Benhuang Yan, Min Jia, Yuan Liu, Shuang Tang, Zhihong Huang, Qiang Fang, Chunmeng Shi, Weibing Li

**Affiliations:** 1 Department of Urology, The Third Affiliated Hospital of Chongqing Medical University, Chongqing, China; 2 Department of Urology, The Southwest Hospital of AMU, Chongqing, China; 3 State Key Laboratory of Trauma, Burns and Combined Injury, Institute of Rocket Force Medicine, Third Military Medical University, Chongqing, China

**Keywords:** bladder smoothmuscle cells, IR-780, lower urinary tract symptoms, nuclear factor erythroid 2-related factor 2 pathway, partial bladder outlet obstruction

## Abstract

**Introduction:**

Partial bladder outlet obstruction (pBOO) is the most common cause of lower urinary tract symptoms (LUTS). Prolonged BOO induces bladder remodeling, which can lead to severe bladder dysfunction and refractory LUTS in some patients, even after obstruction resolution. This condition significantly impairs patients’ quality of life, and no effective treatment is currently available. This study investigated a pBOO rat model using IR-780, a novel near-infrared lipophilic dye with potential targeted antioxidant effects.

**Methods:**

A partial ligation of the rat bladder neck was performed to establish a pBOO model. After confirming successful modeling, the rats were randomly divided into sham, sham + IR-780, pBOO, and pBOO + IR-780 groups (eight rats per group). One week post-surgery, rats received intraperitoneal injections of IR-780 (0.667 mg/kg) or an equivalent volume of phosphate buffered saline solution twice weekly for 3 weeks. Before evaluating efficacy using the bladder filling manometry method, we examined the distribution of IR-780 in tissues and subcellular compartments *via* confocal fluorescence imaging.

**Results:**

IR-780 accumulated at high levels in the bladders of rats with pBOO, where it was primarily taken up by bladder smooth muscle cells (BSMCs) and localized within the mitochondria. Bladder pressure measurements revealed that IR-780 significantly improved bladder function in rats with pBOO. IR-780 effectively mitigated pathological changes in bladder smooth muscle tissue and concurrently alleviated pBOO-induced reflux nephropathy. *In vitro* and *in vivo* experiments confirmed that IR-780 significantly reduced apoptosis in BSMCs. Moreover, cryosection staining and transmission electron microscopy results demonstrated that IR-780 markedly decreased reactive oxygen species levels in BSMCs from rats with pBOO, prevented mitochondrial mass and morphological damage, and significantly reduced the levels of mitochondrial apoptosis pathway-related proteins (Bcl-2, Bcl-2-associated X, cytochrome C, and Caspase-9). We found that IR-780 upregulated the expression of nuclear factor erythroid 2-related factor 2 (Nrf2) and its associated antioxidant proteins in the bladder tissue of rats with pBOO.

**Conclusion:**

IR-780 improved urinary function and complications in rats with pBOO by protecting BSMC mitochondria from oxidative stress, which was potentially mediated through the activation of the Nrf2 pathway.

## 1 Introduction

Bladder outlet obstruction (BOO) is a common urological condition and a frequent cause of lower urinary tract symptoms (LUTS) (Wu et al., 2024). Partial BOO (pBOO) has diverse etiologies, including functional factors such as bladder neck obstruction, pelvic floor muscle hyperactivity, and mechanical factors such as urethral stricture, posterior urethral valves, and benign prostatic hyperplasia (Meier and Padmanabhan, 2016). pBOO leads to urinary dysfunction, detrusor overactivity, vesicoureteral reflux, urinary tract infections, and an overactive bladder, significantly impairing patients’ quality of life (Clout et al., 2025; Solomon et al., 2018; Miyata et al., 2019). In general, relieving obstruction can alleviate LUTS; however, long-term BOO induces structural and functional changes in the bladder, known as bladder remodeling, which result in persistent severe bladder dysfunction and refractory LUTS in some patients, even after the obstruction is resolved (Barbosa et al., 2017; Chen et al., 2021; Hughes et al., 2017). The precise mechanisms underlying pBOO-induced bladder remodeling remain unclear, and no effective clinical treatments are currently available to alleviate bladder dysfunction and its complications secondary to BOO.

Currently, few effective pharmacological therapies exist for bladder injury secondary to pBOO, which may be attributable to the complex pathophysiological mechanisms underlying this condition (Jiang et al., 2021; Miyazaki et al., 2020). Progressive bladder injury caused by pBOO is considered an ordered yet overlapping process, progressing from inflammation to fibrosis (Hughes et al., 2016). Obstruction causes excessive stretching of the bladder wall, increased pressure, and hypoxia/reperfusion within the bladder, progressively disrupting the bladder’s physical structure and function (Metcalfe et al., 2010). Following pBOO, elevated intravesical pressure stimulates the proliferation of bladder smooth muscle cells (BSMCs) as well as proliferation and swelling of urothelial cells. These changes induce inflammation, activate bladder C-fibers, and attenuate voiding-related reflexes, thereby impairing bladder contraction and relaxation. Concurrently, pressure alterations can modulate the expression of bladder receptors, including nicotinic acetylcholine and purinergic receptors (Chen et al., 2012; Wazir et al., 2013), and promote the release of excitatory neurotransmitters such as ATP, leading to increased resistance in the urinary outflow tract and detrusor overactivity (Dunton et al., 2018). Structurally, in addition to the effects of hypoxia in pBOO (Stephany et al., 2013), mechanical traction elevates inflammatory mediators such as nuclear factor kappa-B (NF-κB), tumor necrosis factor (TNF)-α, and interleukin (IL)-6 (Drzewiecki et al., 2012), while ischemia-reperfusion increases oxidative stress in bladder tissue, leading to a surge in reactive oxygen species (ROS) (Miyata et al., 2019). Furthermore, the role of cellular energy metabolism disorders in abnormal tissue remodeling responses has been extensively demonstrated (Hughes et al., 2019). The mechanisms described above contribute to the pathophysiology of pBOO and trigger injury repair mechanisms, leading to the recruitment of neutrophils, monocytes, and other innate immune cells to the injury site to clear cellular debris and pathogens. Simultaneously, they coordinate with fibroblasts, endothelial cells, epithelial cells, and related stem cells to synthesize and release matrix metalloproteinases along with various growth factors and cytokines. Under the influence of pro-repair factors like transforming growth factor (TGF-β), these cells stimulate fibroblast migration and proliferation, promoting their activation into myofibroblasts. These cells then massively release extracellular matrix proteins such as collagen and fibronectin to participate in tissue repair (Eming et al., 2017; Lai et al., 2024). If the injury persists during the repair process, chronic repair may occur, leading to the loss of compensatory mechanisms and pathological organ fibrosis or scar formation (Wynn and Ramalingam, 2012). Mechanical pressure, bladder wall hypoxia, oxidative stress, inflammation, cellular metabolic disorders, and fibrosis contribute to the pathogenesis of pBOO. Although surgical decompression can relieve bladder wall pressure, a therapeutic agent capable of targeting different stages of pBOO is still required. Such an agent must not only possess anti-inflammatory and anti-fibrotic properties but also protect the bladder from oxidative stress damage caused by ischemia-reperfusion injury during the early stages of disease onset, which is a critical requirement.

Nuclear factor-erythroid 2-related factor 2 (Nrf2) is well-studied redox-induced transcription factor (Wardyn et al., 2015) that is normally sequestered in the cytoplasm through interaction with the actin-binding protein Keap 1. Exposure to ROS triggers the release of Nrf2 from Keap 1, allowing it to translocate into the nucleus and stimulate gene transcription (Hassanein et al., 2021). It primarily increases antioxidant enzymes such as superoxide dismutase (SOD), glutathione peroxidase (GPX), and heme oxygenase-1 (HO-1) (Behl et al., 2021), thereby protecting the body against oxidative stress. Nrf2 activation mitigates oxidative damage, inflammation, and cell death in the liver and kidneys (Sacks et al., 2018). Furthermore, recent evidence indicates that activating the Nrf2 signaling pathway can also improve mitochondrial dysfunction in the brains of aged mice (Hong et al., 2025). Previous studies have demonstrated that activation of the Nrf2 pathway can also effectively inhibit bladder tissue damage, particularly diabetic bladder dysfunction (Wang J. et al., 2021).

In a previous study, we synthesized IR-780, a novel near-infrared (NIR) lipophilic dye with seven methyl groups, which demonstrates excellent biocompatibility and minimal cellular contamination in the microenvironment. This fluorescent small-molecule dye exhibits tissue-damage-targeting properties, preferentially accumulates in mitochondria, and protects against various injuries by modulating oxidative stress. Its protective effects have been observed in models of acute lung injury and fibrosis, ischemia-reperfused brain microvascular endothelial cells, and cardiomyocytes (Chen et al., 2024; Luo et al., 2021; Zhang et al., 2023). Additionally, we found that IR-780 localized to the bladder urothelium following bladder instillation in a rat model of radiation cystitis. Concurrently, IR-780 ameliorated both acute urinary tract mucosal injury and late-stage bladder dysfunction induced by radiation cystitis (Li et al., 2023). These results suggest that IR-780 may be a potential imaging and therapeutic agent. However, whether IR-780 can protect bladder function after pBOO remains unclear. The present study investigates the effects of IR-780 on bladder function and pathological changes in rats with pBOO, as well as its potential mechanisms of action.

## 2 Materials and methods

### 2.1 Animals

Female Sprague-Dawley rats were purchased from the animal facility of the Central Animal Breeding Service Center at Army Medical University (AMU, Third Military Medical University), Chongqing, China. All protocols and animal research procedures were approved by the Ethics Committee and conducted in accordance with the guidelines of the Animal Care and Use Committee of the Third Military Medical University. Rats were housed at room temperature under a 12-h light/dark cycle with free access to food and water.

### 2.2 Reagents and materials

The synthesized IR-780 powder was pre-dissolved in dimethyl sulfoxide (DMSO) to a concentration of 10 mmol and stored at −20 °C for later use. Prior to each administration, the solution was diluted 50-fold with sterile PBS and thoroughly mixed. All operations described above were performed in the dark.

### 2.3 Surgery

Anesthetize 8-week-old female Sprague-Dawley rats with isoflurane under surgical conditions and disinfect the suprapubic region. Lubricate and insert a sterile catheter with an outer diameter of approximately 1 mm into the urethra. Make a 1 cm incision in the midline of the lower abdomen with a surgical blade, bluntly dissect the muscle layer, expose the bladder neck and bladder, and ligate the bladder neck with 5–0 silk suture. After tying the knot, the catheter was removed. Rats in the sham group underwent the same surgical procedure without ligation and served as the control group for comparison.

### 2.4 Treatments of rats

**FIGURE 1:**
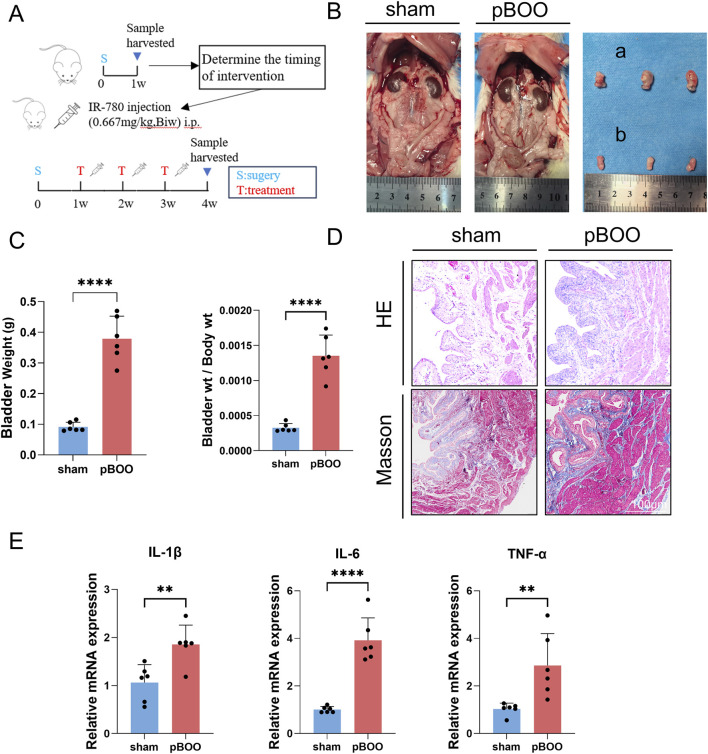
pBOO modeling successfully validated. **(A)** Schematic diagram of the overall rat experimental protocol. **(B)** Left and middle: Rats: Gross images of the urinary tract 1 week after pBOO modeling. Right **(A)** Bladder (partial) of rats in the pBOO group. Right **(B)** Bladder (partial) of rats in the sham group. **(C)** Bladder mass and bladder mass/body weight ratio in sham-operated and pBOO-modeled rats 1 week post-modeling. **(D)** Representative HE-stained and Masson trichrome-stained bladder tissue images from both groups. **(E)** mRNA expression levels of IL-1β, IL-6, and TNF-α in the bladder 1 week after pBOO modeling. Data indicate the mean ± SD (*p < 0.05, **p < 0.01, ***p < 0.001, ****p < 0.0001). Biw: Bis in week.

The experiment comprised two phases: model validation and formal experimentation. During model validation, rats were divided into two groups: sham (control group, n = 6) and pBOO (surgical group, n = 6). One week post-surgery, bladder inflammation, structure, and molecular characteristics were assessed to validate modeling success and determine optimal treatment timing. The formal experimental phase randomly assigned rats to four groups: sham (control group, n = 8), sham + IR-780 (control group + IR-780, n = 8), pBOO (surgical group, n = 8), and pBOO + IR-780 (surgical group + IR-780, n = 8). One week post-surgery, rats received intraperitoneal injections of IR-780 (0.667 mg/kg) or an equivalent volume of PBS solution twice weekly for 3 weeks. This dosage was determined based on the results of our laboratory’s previously published research (Chen et al., 2024). Four weeks after pBOO surgery, each group underwent urodynamic testing. Blood samples were collected from the retroorbital sinus to analyze renal function changes. Rats were then humanely euthanized, and their major organs were harvested for *ex vivo* imaging. Finally, bladder inflammation, structure, molecular biology, and histological features were assessed, along with pBOO-induced reflux nephropathy. The experimental workflow is illustrated in Figure 1A. It is worth noting that the model validation phase and the formal experimental phase utilized two completely independent batches of animal samples. No animal was reused across experiments in different phases.

### 2.5 *Ex Vivo* and In vitro NIR imaging of IR-780

Rats were pretreated with IR-780 (0.667 mg/kg, i.p.) or PBS 24 h prior to near-infrared fluorescence imaging using the Kodak FX Professional Imaging System (New Haven, CT) to detect IR-780 distribution in organs.To examine the distribution of IR-780 in the bladder, bladder tissue was frozen and sectioned into 8 μm slices. IR-780 fluorescence signals were detected using Leica LAS AF Lite software (Leica, Wetzlar, Germany).To determine the subcellular localization of IR-780 in BSMCs, primary BSMCs were isolated from normal SD rats and cultured using the adherent culture method. Immunofluorescence staining with anti-α-SMA antibody (1:200, MB9212S, Abmart) was performed to identify the isolated cells as bladder smooth muscle cells. Cultured BSMCs were then seeded in 35 mm culture dishes and incubated with 20 μM IR-780 for 20 min, followed by incubation with 100 nmol/L MitoTracker Green (C1048, Beyotime, China) for 30 min. Cell nuclei were stained with Hoechst 33,342 (C1022, Beyotime, China) and imaged using a confocal microscope (Leica TCS SP5).

### 2.6 Cystometry In vivo

This study employed cystometry under anesthesia to evaluate urinary function in rats. Intraperitoneal injection of 1.2 g/kg urethane was used to induce anesthesia and immobilize rats 4 weeks post-surgery. Throughout the bladder pressure measurement process, anesthesia depth was assessed periodically *via* toe pinch reflex testing. Appropriate anesthesia was defined as complete absence of pain withdrawal reflexes while maintaining stable baseline vital signs (e.g., spontaneous respiration, heart rate). Based on reflex monitoring results, urethane was micro-titrated according to a pre-established dosing protocol to maintain stable light anesthesia. A constant-temperature heating pad was used throughout the experiment to maintain stable body temperature.A midline abdominal incision was made to expose the bladder, and a PE-50 catheter was inserted into the apex of the bladder dome. The other end of the catheter was connected *via* a three-way connector.One end of the three-way connector was connected to a pressure transducer (Laborie Medical Technologies Inc., Beijing, China) to record bladder pressure, while the other end was connected to an infusion pump. Sterile room-temperature saline was slowly infused at a constant rate of 18 mL/h. Pumping was immediately stopped upon observing urine in the external urethra and resumed once urination ceased. After the bladder pressure curve stabilized, it was automatically recorded by computer for 20 min.Immediately after urination, stop the infusion, withdraw the residual fluid from the bladder, and measure its volume to determine the residual urine volume (RV). In addition to residual urine volume, test parameters include urinary frequency within 20 min, maximum bladder capacity (MBC, volume of saline pumped prior to first voiding), maximum voiding pressure (MVP, peak pressure during the active voiding phase), voiding efficiency, and bladder compliance [BC, calculated as (MBC/TP - BP)]. Threshold pressure (TP) is defined as the intravesical pressure prior to voiding. Baseline pressure (BP) is defined as the minimum pressure between two voiding events.Measurements were taken on four rats per group. The average of three urination cycles per rat was used to eliminate any variation. This procedure was performed three times per rat.

### 2.7 Histological examinations

Each group of rats was euthanized after measuring bladder wet weight. Bladder and kidney tissues were collected and rapidly frozen in liquid nitrogen or immersed in 4% paraformaldehyde. Following fixation and dehydration in 4% paraformaldehyde, tissues were paraffin-embedded, sectioned into 5 μm slices on slides, and subsequently subjected to H&E and Masson’s trichrome staining.

For immunofluorescence analysis of tissue sections, paraffin sections are deparaffinized and rehydrated, then treated with 3% hydrogen peroxide at room temperature for approximately 10 min to inactivate endogenous peroxidase activity.Then, place the sections in a container with antigen retrieval solution, heat in an autoclave until full pressure is reached for 3 min, followed by blocking with 1% goat serum albumin at room temperature for 30 min. Incubate overnight at 4 °C with anti-α-SMA (Abmart, 1:200), then incubate the sections with the corresponding secondary antibody at 37 °C for 1 h. After washing, stain cell nuclei with DAPI (C1006, Beyotime, China). Stained bladder sections were examined under an optical microscope (Olympus Corporation, Tokyo, Japan), and images were captured using a digital camera mounted on the microscope. All images were analyzed using ImageJ software.

### 2.8 Quantitative real‐time PCR (RT‐PCR)

A small portion of bladder tissue from each group was excised for total RNA extraction using Trizol reagent, followed by reverse transcription with the RevertAid First Strand cDNA Synthesis Kit (K1622, Thermo Fisher). Real-time PCR (RT-PCR) was performed using SYBR Green qPCR Master Mix according to the manufacturer’s protocol. Primers are listed in Table 1 (Supplementary Material). Data were normalized to β-actin as an internal control using the ΔCT method.

**TABLE 1:** Primers for quantitative RT-PCR.

Target	Sequence (5′-3′)
IL-1β	Forward:GTGGAGCTTCCAGGATGAGG Reverse:CACACACTAGCAGGTCGTCA
IL-6	Forward:CACTTCACAAGTCGGAGGCT Reverse:TCTGACAGTGCATCATCGCT
Tnfa	Forward:CAGCAGATGGGCTGTACCTT Reverse:AAATGGCAAATCGGCTGACG
Hif1α	Forward:AAGTCAGCAACGTGGAAGGT Reverse:CGGCTGGTTACTGCTGGTAT
IL-10	Forward:GCTCAGCACTGCTATGTTGC Reverse:TTGTCACCCCGGATGGAATG
TGF-β1	Forward:GACTCTCCACCTGCAAGACC Reverse:GGACTGGCGAGCCTTAGTTT
Col1a1	Forward:GTACATCAGCCCAAACCCCA Reverse:CAGGATCGGAACCTTCGCTT
Col3	Forward:ATATGTGTCTGCGACTCGGG Reverse:GGGCAGTCTAGTGGCTCATC
Gapdh	Forward:GACATGCCGCCTGGAGAAAC Reverse:AGCCCAGGATGCCCTTTAGT
Actb	Forward:TGTCACCAACTGGGACGATA Reverse:GGGGTGTTGAAGGTCTCAAA

Col1a1, collagen type I-a1; Col3, collagen type III; Hif1a, hypoxiainducible factor-1a; IL, interleukin; Tnfa, tumor necrosis factor-a; Actb, b-actin.

### 2.9 Western blotting

In brief, extract total protein from each group of bladder tissue/kidney tissue on ice using RIPA buffer containing a mixture of protease and phosphatase inhibitors for 30 min. Depending on protein concentration, prepare samples using 5X loading buffer. Load an equal amount of protein from each sample onto a 4%–12% Tris-glycine SDS-PAGE gel, run electrophoresis, and transfer to a PVDF membrane. Blot the membrane, incubate overnight at 4 °C with the designated primary antibody (1:1000 dilution), and incubate for 1 h with HRP-conjugated secondary antibody (1:2000 dilution). Visualize and analyze band intensities using an enhanced chemiluminescence detection system (Bio-Rad Laboratories) and ImageJ software.

### 2.10 TUNEL staining for detecting apoptosis in bladder tissue

Following the instructions of the detection kit (C1090, Beyotime, China), after dewaxing the sections, add a drop of proteinase K working solution (20 μg/L) onto the tissue sections and incubate for 20 min. Then, add 50 μL of TUNEL mix solution as directed (the negative control group was treated only with 50 μL of fluorescein-labeled dUTP solution). Place the slides in a humid chamber and incubate at 37 °C in the dark for 1 h. Finally, add DAPI-containing anti-fluorescence quenching mounting medium and mount the slides. Imaging of stained sections was performed using a fluorescence microscope.

### 2.11 *In situ* detection of mitochondrial and superoxide levels

Fresh bladder tissue from each group was prepared into frozen sections and incubated briefly at 37 °C in the dark with Mito-Tracker Red, 2′,7′-dichlorodihydrofluorescein diacetate (DHE, 10 μM, Beyotime) and MitoSOX™ Red (10 μM, Invitrogen) for 30 min at 37 °C in the dark. This measured the relative number of active mitochondria in BSMCs, the *in situ* levels of intracellular ROS, and mitochondrial superoxide levels, respectively. Fluorescence intensity was quantified using ImageJ software.

### 2.12 Transmission electron microscopy

Each group of fresh bladder smooth muscle tissue was trimmed into 1 × 1 × 3 mm tissue blocks, fixed overnight in 4% glutaraldehyde, fixed with 1% osmium tetroxide, dehydrated in graded ethanol, and embedded in fresh 100% resin. Ultrathin sections cut from the blocks were stained with 3% uranium acetate in saturated alcohol and lead citrate for 8 min each, then examined by transmission electron microscopy at 100 kV. In all groups, bladder tissue samples for TEM analysis were uniformly obtained from the mid-layer smooth muscle region of the bladder body.

### 2.13 Statistical analysis

Each experiment was repeated at least three times. All statistical analyses were performed using SPSS software version 26.0 and GraphPad Prism software version 10.0. Pairwise comparisons were conducted using one-way or two-way analysis of variance (ANOVA) to determine statistical significance. P < 0.05 was considered statistically significant.

## 3 Results

### 3.1 Successful validation of rat modeling

Seven days after surgery, rats in the pBOO group exhibited markedly distended and swollen bladders with slightly dilated ureters (Figure 1B). Upon removal, the bladders in the pBOO groups showed increased weight compared to those in the control groups and displayed macroscopically evident inflammatory changes (Figures 1B,C). Hematoxylin and eosin (HE) staining revealed thickened urothelium and increased inflammatory cell infiltration in the bladders of rats with pBOO. Masson’s trichrome staining demonstrated a thickened muscularis propria without significant collagen deposition. The primary histological features at this stage were inflammation and detrusor muscle hypertrophy (Figure 1D). We assessed the levels of proinflammatory cytokines in the bladder and found that pBOO group exhibited significantly elevated mRNA expression of IL-1β, IL-6, and TNF-α following obstruction (Figure 1E). These results confirm that the pBOO model was successfully established and is suitable for subsequent drug intervention studies.

### 3.2 Biodistribution and accumulation of IR-780

**FIGURE 2:**
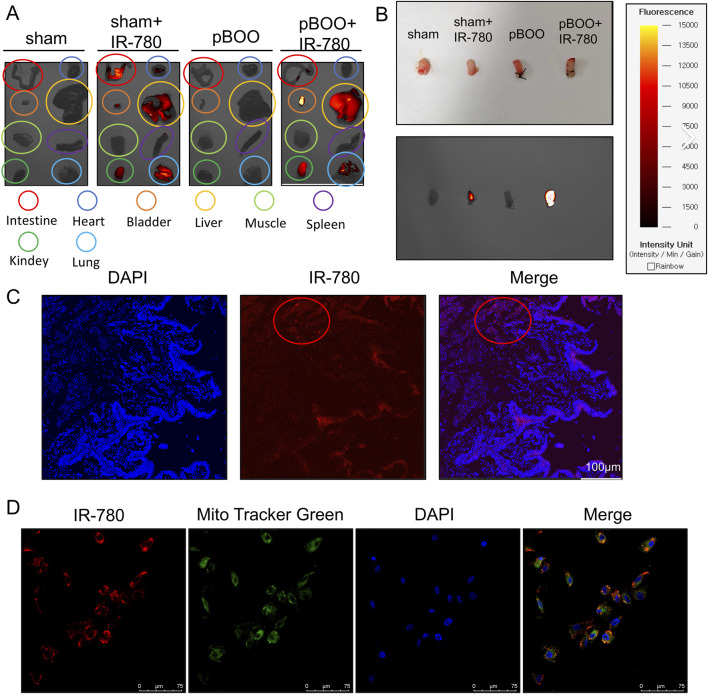
Biodistribution and Subcellular Localization of IR-780. **(A)** *In vivo* NIR imaging of major rat organs 24 h prior to dissection, following 4 weeks of pBOO modeling *via* intraperitoneal injection of IR-780/PBS. **(B)** *In vitro* NIR imaging of bladder tissue from each group (uniform contrast). **(C)** Fluorescence imaging of IR-780 (red) in bladder tissue, scale bar 100 μm. **(D)** Mitochondrial targeting of IR-780 in BSMCs determined by co-staining with IR-780 and Mito-Tracker Green. scale bar 75 μm.

We obtained key organs from the four rat groups for *in vivo* imaging. As expected, in the longitudinal comparison of organs within the same group, IR-780 demonstrated targeting properties in partially obstructed bladders. We observed significantly stronger NIR fluorescence in the bladder than in other organs such as the heart, liver, spleen, lungs, and intestines (Figure 2A). In a cross-group comparison, the pBOO + IR-780 group exhibited markedly higher NIR fluorescence in the bladder than the other three groups (Figure 2B).

To determine the distribution of IR-780 within the bladder wall, histological analysis was performed on fresh-frozen bladder sections obtained 1 day after the intraperitoneal injection of IR-780. Confocal microscopy revealed that IR-780 was primarily localized within the smooth muscle layer of the bladder (Figure 2C). Therefore, this study focused on exploring the role of IR-780 in BSMCs.

To determine the subcellular localization of IR-780, we isolated and cultured primary BSMCs from 8-week-old Sprague-Dawley rats. The cells were identified by immunofluorescence staining for α-smooth muscle actin (α-SMA), a specific smooth muscle cell marker (Supplementary Figure S1). As previously reported, co-localization of IR-780 with MitoTracker Green confirmed its targeted accumulation within BSMC mitochondria (Figure 2D). Collectively, these findings demonstrate that IR-780 can serve as a target dye for pBOO bladders.

### 3.3 IR-780 improves bladder voiding function in pBOO

**FIGURE 3:**
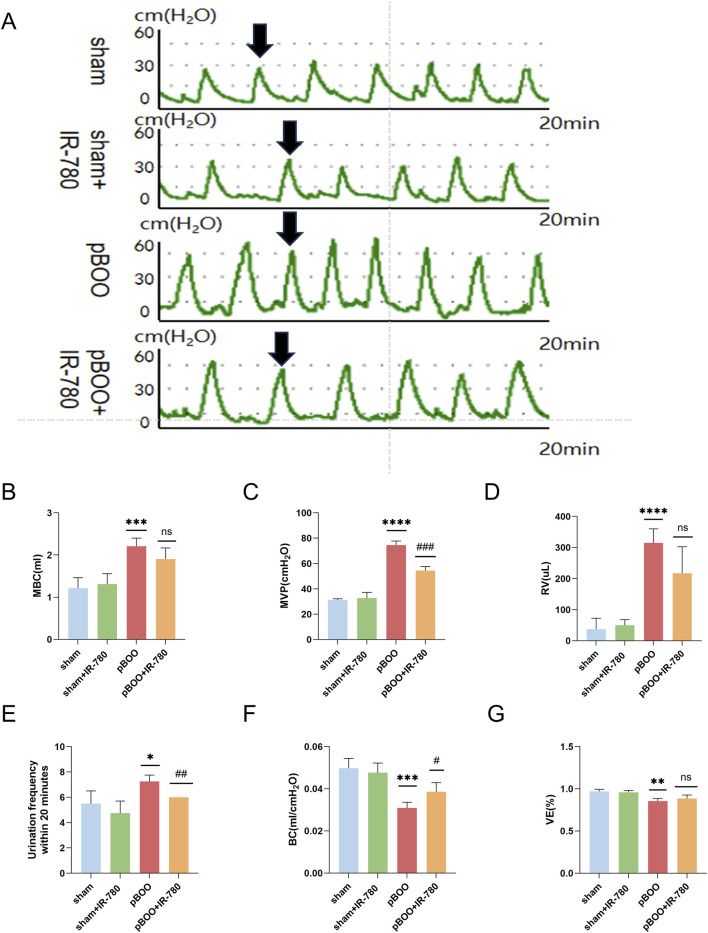
Administration of IR-780 improves bladder dysfunction in pBOO rats (n = 4). **(A)** Representative intravesical pressure curves for rats in each group. **(B)** Maximum bladder capacity (MBC). **(C)** Maximum voiding pressure (MVP). **(D)** Residual volume (RV). **(E)** Frequency of urination in 20 min. **(F)** Bladder compliance [BC; calculated as MBC/(TP-BP)]. **(G)** Voiding efficiency {VE, calculated as [(MBC−RV)/MBC] × 100%}. Data indicate the mean ± SD (ns: p > 0.05, *p < 0.05, **p < 0.01, ***p < 0.001, ****p < 0.0001 vs. sham group; #p < 0.05, ##p < 0.01, ###p < 0.001 vs. pBOO group). Arrows indicate peak voiding.

Four weeks postoperatively, bladder pressure was measured in the rats, and the pressure curves are shown in Figure 3A. Analysis of the bladder pressure-time curves revealed that, compared with rats in the sham group, those in the pBOO group exhibited significantly increased MVP, MBC, RV, and voiding frequency (p < 0.0001, p < 0.001, p < 0.0001, p < 0.05), but decreased BC and VE (p < 0.001, p < 0.01), indicating typical pBOO decompensation. In contrast, the pBOO + IR-780 group showed decreased MVP, voiding frequency and increased compliance (p < 0.001, p < 0.01, p < 0.05 compared to pBOO group), and demonstrated certain improvements despite no significant differences in MBC, RV, or VE (Figures 3B–G). These data confirm that IR-780 administration exerts a protective effect by alleviating voiding-phase and storage-phase dysfunction in pBOO.

### 3.4 Effects of IR-780 on several key cytokines in pBOO rat bladder tissues

The mechanism of pBOO progression involves an ordered overlapping sequence from inflammation to fibrosis (Hughes et al., 2016). Multiple molecular mechanisms are involved. IL-6 and TNF-α, which are classic proinflammatory cytokines, mediate the activation and regulation of immune responses (He et al., 2021; Gheinani et al., 2017). The TGF-β1/Smad 3 pathway is closely associated with organ fibrosis (Meng et al., 2016), while the hypoxia-inducible factor-1α(HIF-1α) pathway represents an adaptive respond to hypoxic environments (Sun et al., 2023). Conversely, IL-10 suppresses proinflammatory responses and limits tissue damage caused by excessive inflammation (Ouyang et al., 2011).

**FIGURE 4:**
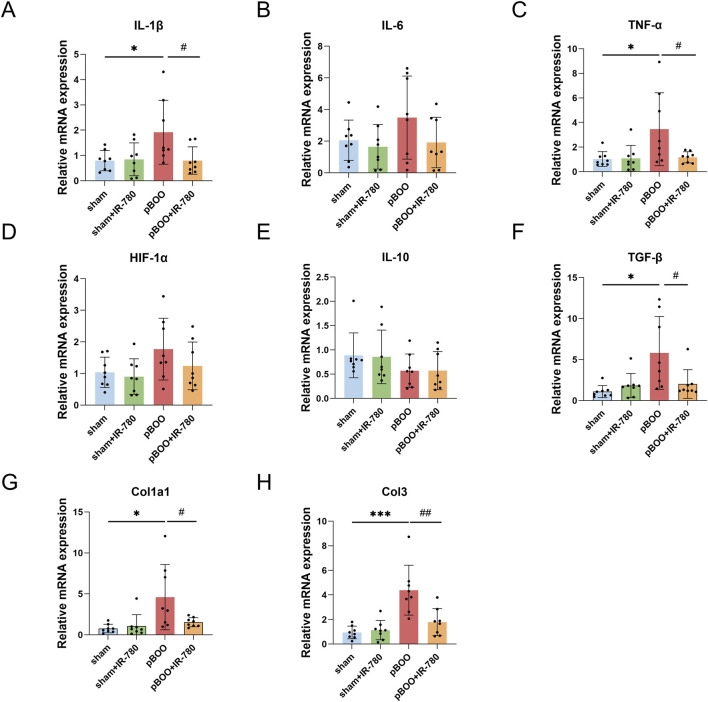
Expression of collagen and cytokines in rat bladders after 4 weeks (n = 8). Quantitative RT-PCR was used to detect IL-1β **(A)**, IL-6 **(B)**, TNF-α **(C)**, hypoxia-inducible factor (HIF)-1α **(D)**, IL-10 **(E)**, transforming growth factor (TGF)-β1 **(F)**, collagen type I-α1 (Col1α1; **(G)**, and collagen type III (Col3; **(H)** groups. Data indicate the mean ± SD (*p < 0.05, **p < 0.01, ***p < 0.001, vs. sham group; #p < 0.05, ##p < 0.01, vs. pBOO group). No statistical significance was found between unmarked groups.

We therefore examined the key molecules associated with pBOO to investigate the effects of IR-780 on several critical cytokines in the bladder tissues of rats with pBOO 4 weeks post-obstruction. In the pBOO group, both IL-1β and TNF-α expression in the bladder were significantly upregulated at the mRNA level (both p < 0.05 compared to the sham group). Following IR-780 treatment, IL-1β and TNF-α expression levels reduced (both p < 0.05 compared to the pBOO group) (Figures 4A,C). The bladder fibrosis factors TGF-β1, Collagen type I α1 chain (Col1a1), and Collagen type III (Col3) were significantly increased in the pBOO group (p < 0.05, p < 0.05, p < 0.001 compared to the sham group), and IR-780 reduced this expression (p < 0.05, p < 0.05, p < 0.01 compared to the pBOO group) (Figures 4F–H). These findings indicate that IR-780 exhibits anti-inflammatory effects and mitigates progressive fibrosis in pBOO-induced bladders.

### 3.5 IR-780 alleviates damage to bladder tissue structure caused by BOO

**FIGURE 5:**
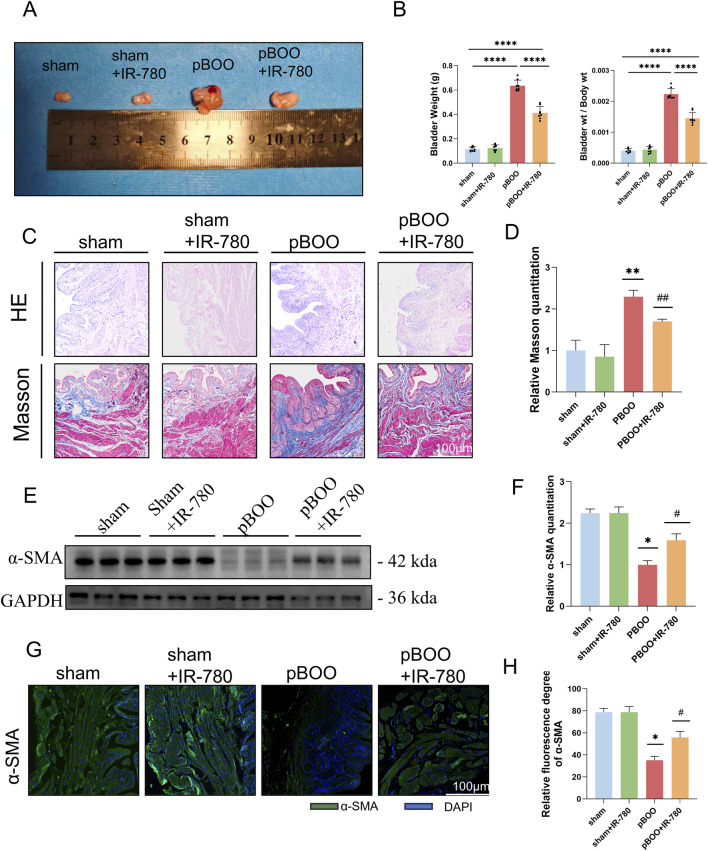
IR-780 ameliorates pBOO-induced bladder histopathological changes. **(A)** Gross pathological images of rat bladders at 4 weeks post-pBOO modeling. **(B)** Bladder weight and bladder weight/body weight ratio in rats from each group 4 weeks after pBOO modeling (****p < 0.0001). **(C)** Representative HE-stained and Masson trichrome-stained images of bladder tissue from each group, scale bar 100 μm. **(D)** Quantitative analysis of collagen fiber levels in Masson staining. **(E,F)** Western blot detection of α-SMA levels in bladder tissue. **(G,H)** Immunofluorescence detection of α-SMA levels in bladder tissue. Data indicate the mean ± SD (*p < 0.05, **p < 0.01, ***p < 0.001, ****p < 0.0001 vs. sham group; #p < 0.05, ##p < 0.01 vs. pBOO group).

In the early stages of BOO, mechanical stress induces the upregulation of α-SMA expression to enhance detrusor contractility, representing compensatory remodeling. However, when obstruction persists long-term, bladder wall fibrosis progressively worsens. Massive smooth muscle cell apoptosis occurs and is replaced by collagen fibers, whereas myofibroblast activation and proliferation enter an exhausted phase, leading to a significant decline in α-SMA synthesis capacity. At this point, the reduced α-SMA levels indicate that the bladder has transitioned from the compensatory phase to the decompensatory phase (Metcalfe et al., 2010; Salinas et al., 2004; Yoshida and Yamaguchi, 2014). After 4 weeks, the bladders in the pBOO group exhibited marked congestion and swelling, with increased weight compared to those in the sham group. However, the bladders in the pBOO + IR-780 group showed slightly reduced volume and weight relative to those in the pBOO group (Figures 5A,B). Compared with the control group, the surgical group exhibited thickened urothelium and inflammatory cell infiltration and progressive changes in the muscularis propria. Muscle bundles gradually thickened, then underwent disruption and dissolution, with widening of the interstitial spaces and subsequent structural disorganization. After 3 weeks of IR-780 intervention, the degree of damage to the bladder tissues showed significant improvement (HE staining) (Figure 5C). Masson’s trichrome staining revealed significantly increased collagen fiber deposition in the pBOO group compared to that in the sham group (P < 0.01). Although collagen deposition remained higher in the pBOO + IR-780 group than that in the control group, it significantly reduced in the pBOO + IR-780 group compared to the pBOO group. (P < 0.01) (Figures 5C,D). Additionally, we detected α-SMA expression in bladder tissues *via* Western blotting and immunofluorescence staining. Decreased α-SMA expression was observed in the pBOO group (vs.control group, p < 0.05), whereas the pBOO + IR-780 group showed increased α-SMA expression (vs.pBOO group, p < 0.05) (Figures 5E–H). This confirms the earlier emphasis on BSMCs as the focus of this study and indicates that IR-780 can restore the reduced smooth muscle content induced by pBOO.

### 3.6 IR-780 ameliorates pBOO-induced kidney injury

**FIGURE 6:**
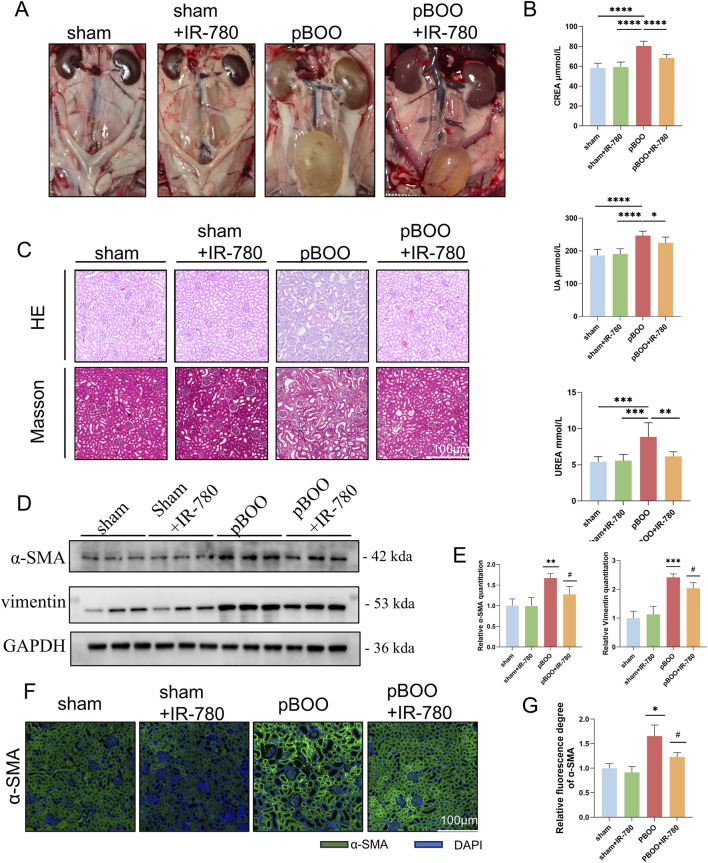
IR-780 treatment reduced PBOO-induced renal injury. **(A)** Gross anatomical images of the urinary tract in rats from each group at 4 weeks post-IR-780 or PBS treatment following sham/surgery. **(B)** Biochemical parameter analysis in rats across groups (*p < 0.05, **p < 0.01, ***p < 0.001, ****p < 0.0001). **(C)** Representative HE-stained and Masson trichrome-stained renal tissue images from each group. **(D,E)** Western blot detection of α-SMA and vimentin protein expression in bladder tissue. **(F,G)** Immunofluorescence detection of α-SMA levels in kindey tissue. Data indicate the mean ± SD (*p < 0.05, **p < 0.01, ***p < 0.001, vs. sham group; #p < 0.05, vs. pBOO group).

BOO ultimately leads to severe complications such as vesicoureteral reflux, bladder dysfunction, bilateral renal damage, renal failure, bladder cancer, and bladder neck tumors (Cisek, 2017). Owing to the varying duration and progression of BOO lesions, many patients still develop progressive bladder dysfunction and renal impairment after surgery, which are difficult to treat (Wang et al., 2023). Therefore, we investigated the effects of IR-780 on pBOO-induced renal injury. Surprisingly, compared to sham rats without ureteral reflux, rats in the pBOO group exhibited markedly dilated ureters and renal pelvises, enlarged kidneys, and increased capsular tension, indicating the presence of vesicoureteral reflux. In contrast, the pBOO + IR-780 group showed a milder presentation: although the ureters were dilated, both kidneys exhibited relatively normal coloration with no significant hydronephrosis or perinephric fluid accumulation (Figure 6A). Serum was extracted from rats to measure serum creatinine (CREA), uric acid (UA), and urea levels. Compared to the sham group, the pBOO group exhibited significantly elevated CREA, UA, and UREA levels. However, IR-780-treated rats with pBOO exhibited reduced CREA, UA, and urea levels (p < 0.0001, p < 0.05, p < 0.01, compared to the pBOO group) (Figure 6B). HE and Masson staining revealed normal glomerular morphology without tubular dilatation in the sham group. The pBOO group exhibited glomerular deformation and atrophy, accompanied by tubular injury and marked glomerulosclerosis (Figure 6C). The pBOO group demonstrated the highest expression of α-SMA and vimentin protein using Western blotting (p < 0.01 and p < 0.001 compared to the sham group). The pBOO + IR-780 group α-SMA showed lower expression than the pBOO group but higher expression than the sham and sham + IR-780 groups; the pBOO + IR-780 group vimentin protein showed lower expression than the pBOO group (p < 0.001, p < 0.001 compared to the pBOO group), but higher expression than the sham and sham + IR-780 groups (p < 0.05 compared to the pBOO group) (Figures 6D,E). Immunofluorescence staining validated these findings (Figures 6F,G), suggesting that IR-780 may suppress pBOO-induced renal fibrosis. In summary, IR-780 treatment ameliorated pBOO-induced renal injury, potentially by reducing bladder injury or through direct protective effects on renal tissue.

### 3.7 IR-780 reduces the apoptosis of BSMCs

**FIGURE 7:**
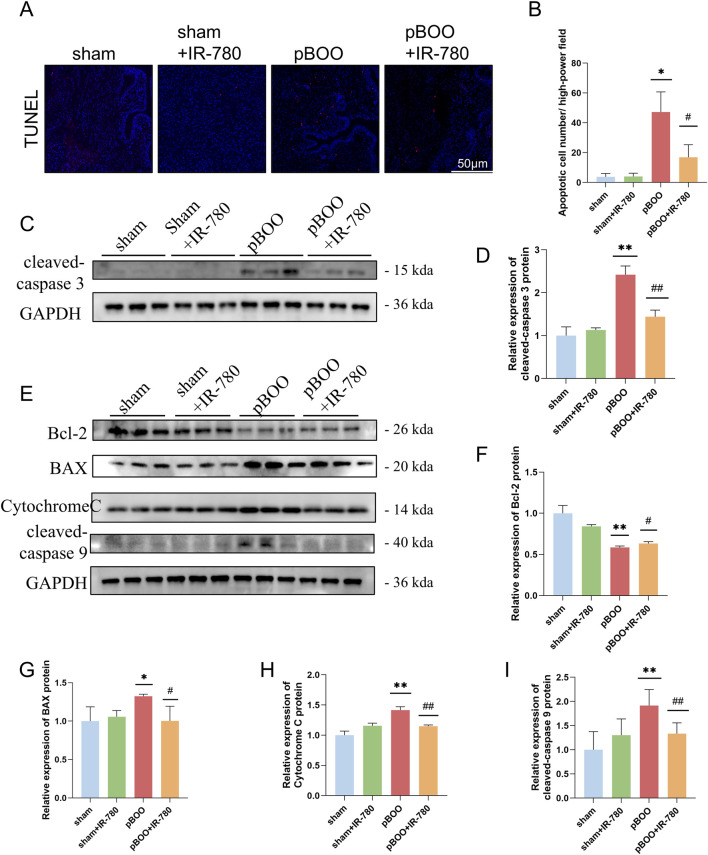
IR-780 reduced the number of apoptotic BSMCs in pBOO and affected mitochondrial apoptosis-related proteins. **(A)** Representative images of TUNEL staining (red) in bladders from each group. **(B)** Number of apoptotic BSMCs per high-power field. **(C,D)** Western blot detection of apoptotic protein cleaved-caspase three levels. **(E–I)** Western blot detection and quantitative analysis of Bcl-2, Bcl-2-associated X, Cytochrome C, and cleaved-caspase 9 protein expression levels in each group. Data indicate the mean ± SD (*p < 0.05, **p < 0.01, ***p < 0.001, vs. sham group; #p < 0.05, ##p < 0.01, vs. pBOO group).

TUNEL staining revealed a significant increase in apoptotic cells within the bladder smooth muscle of the pBOO group compared with the sham and pBOO + IR-780 groups (all P < 0.05). However, apoptosis was markedly reduced in the pBOO + IR-780 group (P < 0.05 compared to the pBOO group) (Figures 7A,B). Western blotting revealed that the protein levels of cleaved Caspase-3 were significantly elevated in the pBOO group compared to the sham group (P < 0.01), whereas they were markedly reduced in the pBOO + IR-780 group compared to the pBOO group (P < 0.01) (Figures 7C,D). These data indicate that IR-780 effectively ameliorates apoptosis in BSMCs from rats with pBOO. Based on the mitochondrial localization of IR-780 in BSMCs, we further examined proteins associated with the mitochondrial apoptotic pathway to investigate the relationship between IR-780 and apoptosis. Western blot analysis revealed that Bcl-2-associated X, Cytochrome C, and cleaved Caspase-9 significantly increased (p < 0.05, p < 0.01, p < 0.01 compared to the sham group), whereas Bcl-2 protein expression decreased (p < 0.01 compared to the sham group). However, IR-780 treatment blocked these changes (Figures 7E–I). Collectively, these data indicate that IR-780 inhibits mitochondrial-related apoptosis in the bladder tissues of rats with pBOO.

### 3.8 IR-780 alleviates mitochondrial damage and ROS production in rats with pBOO

**FIGURE 8:**
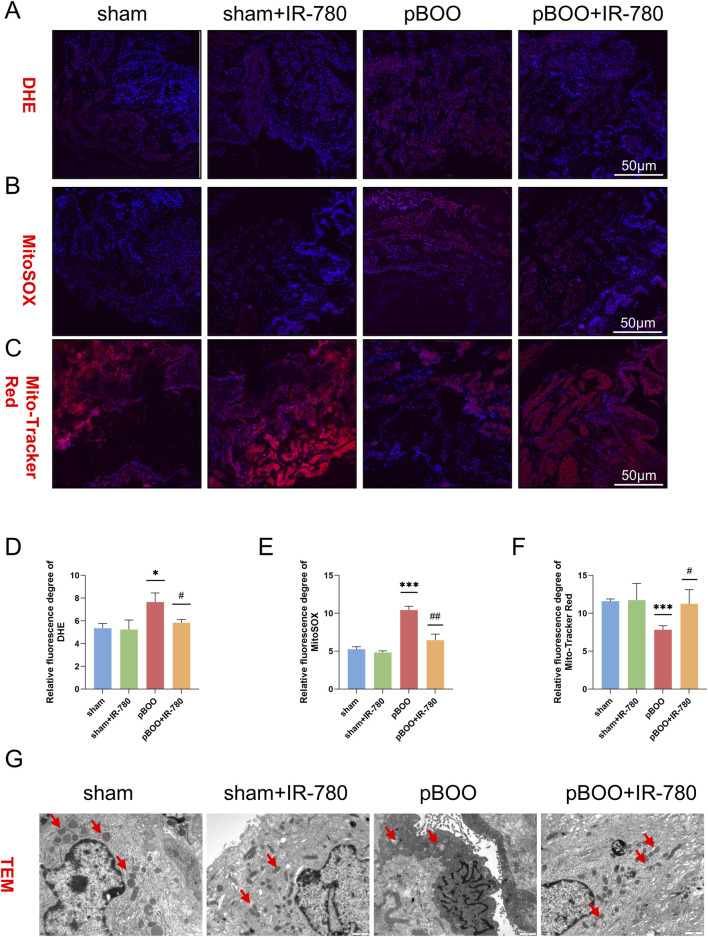
IR-780 mitigates pBOO-induced oxidative stress and mitochondrial damage in BSMCs. **(A,D)** Intracellular ROS in the bladder detected by DHE staining of frozen bladder sections and quantified by fluorescence intensity analysis, scale bar 50 μm. **(B,E)** Quantitative analysis of mitochondrial ROS in the bladder detected by MitoSOX staining of frozen bladder sections, with fluorescence intensity, scale bar 50 μm. **(C,F)** Quantitative analysis of mitochondrial mass in BSMCs measured by Mito-Tracker Red staining of frozen bladder tissue sections, with fluorescence intensity, scale bar 50 μm. **(G)** Representative transmission electron micrographs of mitochondrial morphology and structure in BSMCs. Red arrows indicate mitochondria. Data indicate the mean ± SD (*p < 0.05, **p < 0.01, ***p < 0.001, vs. sham group; #p < 0.05, ##p < 0.01, vs. pBOO group).

Frozen sections of bladder tissues from rats in each group were stained with DHE and MitoSOX to measure the cellular and mitochondrial superoxide levels. Quantitative analysis of DHE and MitoSOX staining revealed significantly elevated levels of cellular and mitochondrial superoxides in the pBOO group (P < 0.05 and P < 0.001 compared to the sham group), whereas IR-780 treatment suppressed the elevation in cellular and mitochondrial superoxide levels (P < 0.05 and P < 0.01 compared to the pBOO group) (Figures 8A,B,D,F). This demonstrates that IR-780 possesses certain antioxidant effects. Fluorescent staining with MitoTracker Red was used to assess mitochondrial damage in bladder tissues of each rat group. The results showed that the number of viable mitochondria in the bladder tissues of the pBOO group significantly reduced (P < 0.001 compared to the sham group), whereas the number of mitochondria increased after IR-780 treatment (P < 0.05 relative to the pBOO group) (Figures 8C,F). Transmission electron microscopy images revealed changes in mitochondrial structure and morphology. Cells in the sham group exhibited a higher number of mitochondria with normal morphology, showing a well-organized arrangement and a clear structure. In contrast, cells in the pBOO group demonstrated a reduced mitochondrial quantity with significantly diminished, fractured, and even dissolved cristae. Numerous mitochondria exhibited calcification and swelling, accompanied by decreased electron density in the mitochondrial matrix and vacuolar degeneration. Compared to the surgery group, the pBOO + IR-780 group exhibited a significant increase in mitochondrial quantity and cristae number, with the morphology approaching normal (Figure 8G). These findings indicate that IR-780 exerts a protective effect on mitochondria within BSMCs.

### 3.9 IR-780 participates in the Nrf2 pathway activation

**FIGURE 9:**
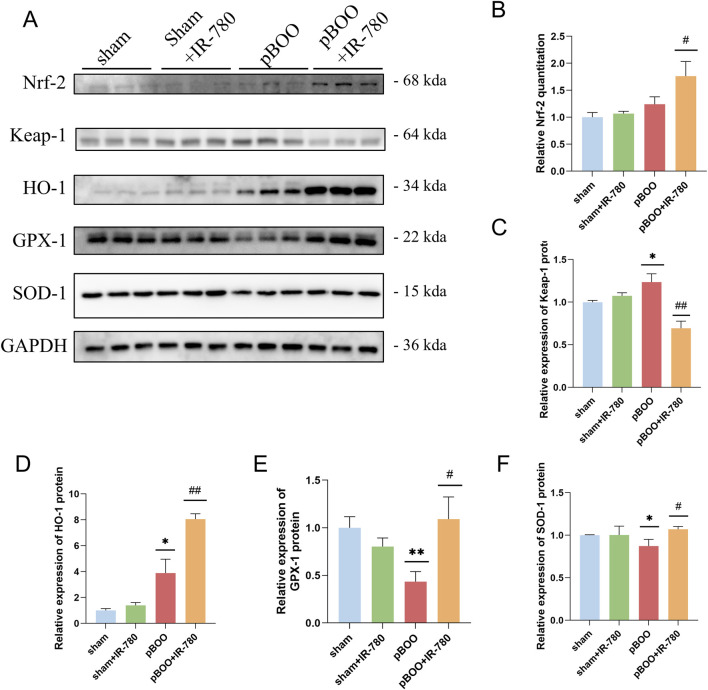
IR-780 activates Nrf2 and increases antioxidant-related protein levels. **(A–F)** Western blot analysis and quantitative analysis of Nrf-2, Keap-1, HO-1, GPX-1, and SOD-1 in bladder tissues from each group. Data indicate the mean ± SD (*p < 0.05, **p < 0.01, vs. sham group; #p < 0.05, ##p < 0.01, vs. pBOO group).

**FIGURE 10:**
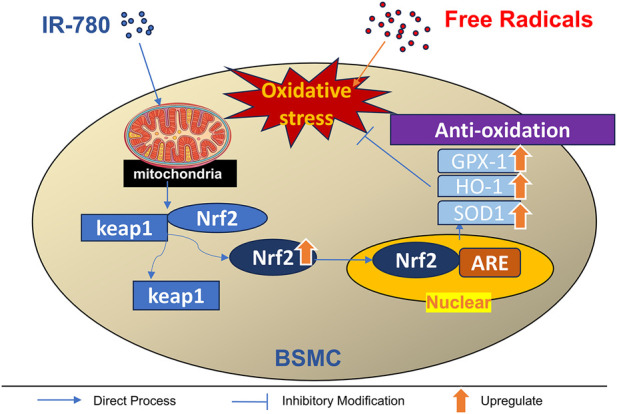
A simple diagram of the drug’s mechanism of action.

Based on the above results, we further investigated whether the antioxidant effects of IR-780 were mediated by Nrf2 activation. Western blotting analysis of representative proteins in four bladder groups revealed that compared to the sham group, the pBOO group exhibited decreased levels of HO-1, GPX1, and SOD1 (p < 0.05, p < 0.01 and p < 0.05) (Figures 9A,B,D–F), and increased Keap1 levels (p < 0.05) (Figures 9A,C). However, IR-780 treatment upregulated pBOO-induced Nrf2 protein expression (vs. pBOO group p < 0.05) while downregulating Keap1 (p < 0.01), reversing the decrease in the antioxidant proteins HO-1, GPX1, and SOD1 (p < 0.01, p < 0.05, and p < 0.05). These results suggest that the protective effect of IR-780 may be related to Nrf2 activation (Figure 10).

## 4 Discussion

Bladder dysfunction caused by BOO is becoming an increasing concern among clinicians (Gravas et al., 2018). By 2018, approximately 1.1 billion people worldwide had experienced LUTS due to benign prostatic hyperplasia, with Asian populations potentially being the most severely affected (Irwin et al., 2011). However, there is no sufficiently effective clinical treatment for pBOO, as most current therapies are designed solely to alleviate symptoms rather than delay or reverse the progressive fibrotic damage associated with the condition (Lai et al., 2024). Therefore, there is an urgent need to develop effective drugs to maintain normal bladder function and prevent or slow the progression of pBOO. Owing to BOO, localized pressure increases within the bladder wall caused by traction, which activates the ischemia-reperfusion mechanism (oxidative stress). Oxidative stress damage is considered one of the core mechanisms underlying the pathogenesis and progression of pBOO (Miyata et al., 2019). In previous studies, we demonstrated that IR-780 exhibits targeted localization to mitochondria and possesses antioxidant properties in cell therapy. In this study, we investigated whether IR-780 could mitigate the progression of bladder dysfunction and its related complications in rats with pBOO.

Rats with BOO may develop bladder dysfunction 4 weeks post-surgery, characterized by symptoms of detrusor overactivity—such as increased urinary frequency, shortened voiding intervals, and reduced urinary output—along with elevated oxidative stress, bladder smooth muscle fibrosis, and hypertrophy (Ozturk et al., 2020). Therefore, we selected the 4-week mark as the experimental endpoint. To distinguish between the therapeutic and preventive effects of the drug, we initiated treatment 1 week post-surgery and validated the model using rats at the same time point. Through gross dissection, histopathological staining, and detection of inflammatory factor mRNA, we successfully established the pBOO rat model, providing the foundation for subsequent studies.

Before evaluating the efficacy of IR-780 in improving pBOO, we first assessed its distribution across various organs, within different layers of the bladder wall and at the subcellular level. IR-780 exhibited the highest fluorescence intensity in damaged bladder tissues and accumulated in the smooth muscle layer of the bladder wall, with minimal involvement in other layers. Therefore, BSMCs are the primary focus of the present study. Bladder function was altered following BOO. Detrusor smooth muscle hypertrophy compensates for the increased muscle contractility required to overcome increased urethral resistance during urination (Fusco et al., 2018). Using primary cell isolation techniques, we extracted BSMCs and simultaneously observed the accumulation of IR-780 within BSMC mitochondria, which is consistent with the results of previous studies. Mitochondria serve as the primary endogenous sources of ROS within cells. The energy from the electrons interacts with water to form superoxide radicals, which are then converted by SOD into peroxides and ultimately generate ROS. In other words, mitochondria are the principal generators of ROS (Del Re et al., 2019). The mechanism of oxidative damage induced by pBOO aligns with the target site of IR-780, leading us to speculate whether this mitochondria-targeted small-molecule drug could be used to treat such diseases. Our preliminary research demonstrates that IR-780 or its derivatives accumulate in damaged bladders and treat urinary dysfunction and complications through their antioxidant and anti-apoptotic effects (Wang J. et al., 2021; Li et al., 2023), suggesting that IR-780 may be a potential therapeutic agent for pBOO.

Functional assessments of rats with pBOO revealed significant increases in MVP, MBC, RV, and voiding frequency in rats with pBOO, accompanied by decreases in BC and VE. These findings indicate impaired bladder function and progression to the decompensation phase of pBOO. Conversely, the pBOO + IR-780 group exhibited reduced MVP and voiding frequency with enhanced compliance, demonstrating that IR-780 intervention improved urinary function in rats. At the molecular level, RT-PCR analysis of key factors involved in pBOO progression revealed significantly upregulated mRNA expression of inflammatory mediators IL-1β and TNF-α, alongside increased expression of fibrotic factors TGF-β1, Col1a1, and Col3. IR-780 treatment reduced this expression pattern. Therefore, we conducted further investigations into inflammatory and fibrotic damage. At the histological level, pathological staining and Western blot experiments revealed that IR-780 improved fibrosis in rat bladder smooth muscles. These results demonstrate that IR-780 ameliorates bladder dysfunction and fibrotic progression induced by pBOO. Furthermore, we observed that IR-780 treatment enhanced resistance to apoptosis in bladder smooth muscle tissue. This aligns with the reduced smooth muscle content and decompensation observed in the bladder walls of rats with pBOO, in which apoptosis was associated with mitochondrial dysfunction.

Notably, this study unexpectedly found that IR-780 ameliorates pBOO-induced renal injury, manifested by reduced CREA, UA, and UREA levels, along with lessened renal histopathological damage. Based on the core findings of this study and relevant literature reports, we hypothesize that its renal protective effects may be mediated through a dual “direct-indirect” pathway. On one hand, as a key discovery of this study, IR-780 significantly alleviates pBOO-induced oxidative stress and inflammatory infiltration in the bladder wall, thereby improving bladder outlet obstruction. Once bladder outlet obstruction is relieved, the mechanical pressure load on the upstream kidneys from the lower urinary tract is significantly reduced, alleviating renal interstitial congestion, edema, and tubular injury. This represents the indirect pathway through which IR-780 exerts renal protection and constitutes a clearly identifiable potential regulatory mechanism in this study. On the other hand, we cannot exclude the direct protective effects of IR-780 on renal tissue: IR-780 can be distributed throughout the body *via* the blood circulation to various tissues and organs. Previous studies have confirmed that in organ injury models such as the lungs and heart, it exerts antioxidant and anti-inflammatory protective effects by directly scavenging reactive oxygen species, inhibiting the expression of inflammatory factors (such as TNF-α and IL-6), and reducing apoptosis (Chen et al., 2024; Luo et al., 2021). Based on this, it is speculated that IR-780 may directly act on renal tissue in a similar manner to mitigate pBOO-induced renal parenchymal injury. Future research will focus on this scientific question, further validating IR-780s direct regulatory effects on renal tissue. This will clarify whether it functions by modulating kidney-specific oxidative stress and inflammation-related signaling pathways (such as the Nrf2/HO-1 and NF-κB pathways), thereby fully elucidating the molecular mechanisms underlying IR-780s renal protective effects.

Prolonged and chronic cyclic ischemia/reperfusion generates ROS. Elevated ROS levels induce oxidative stress in the bladder and play a significant role in bladder dysfunction through alterations in cellular and molecular characteristics, including vascular and nerve density, as well as fibrosis induction (Kalorin et al., 2008; Nomiya et al., 2012). ROS and pBOO are associated with bladder dysfunction, as they directly damage detrusor muscle mitochondria, thereby inhibiting energy production and impairing detrusor contractility (Baird and Yamamoto, 2020). Bratslavsky et al. demonstrated that in rat models, ROS-mediated reperfusion events inflicted greater damage than isolated ischemic events (Bratslavsky et al., 2003). Based on this evidence, ROS are considered the primary pathogenic factor responsible for bladder dysfunction caused by pBOO (Miyata et al., 2019). Therefore, we focused our research on mitochondria and oxidative stress. In this study, we found that the levels of total intracellular and cell-derived ROS in the bladder tissues of rats with pBOO were significantly higher than those in control rats, and these levels were maintained after IR-780 treatment. Furthermore, MitoTracker Red fluorescence detection and transmission electron microscopy revealed markedly reduced mitochondrial number and activity, along with mitochondrial structural disruption, in BSMCs of rats with pBOO. These data indicate that bladder tissue damage in rats with pBOO may result from persistent oxidative stress, and IR-780 ameliorated these changes.

Nrf2/Keap1 is the most important transcriptional mechanism regulating antioxidant genes and maintaining cellular redox homeostasis (Motohashi and Yamamoto, 2004). The Nrf2 pathway improves bladder dysfunction in cyclophosphamide-induced cystitis by suppressing oxidative stress (Ni et al., 2021). Liu et al. used sulforaphane to activate the Nrf2–antioxidant response element (Nrf2–ARE) pathway and improve bladder dysfunction in a rat model of pBOO (Liu et al., 2016). In the present study, our data indicate that IR-780 intervention upregulated Nrf2 protein expression. In the pBOO + IR-780 group, the expression of antioxidant proteins HO-1, GPX1, and SOD1 also significantly increased; this upregulation can mitigate or counteract oxidative stress-induced damage to BSMCs and their mitochondria. In summary, IR-780 reduces oxidative stress levels in bladder smooth muscle tissue by activating the intracellular Nrf2/Keap-1 signaling pathway and promoting the expression of a series of endogenous antioxidant proteins. This may represent a potential mechanism underlying its protective effects against oxidative stress damage in BSMCs and their mitochondria, as well as the improvement of urinary function in rat with pBOO.

This study has certain limitations: First, there is no standard definition for the time point at which pBOO progresses from the compensatory phase to the decompensatory phase. Through preliminary experiments, we found that rats exhibited urodynamic changes indicative of the decompensatory phase at 4 weeks post-surgery. Therefore, we set 4 weeks post-surgery as the endpoint for this study. Second, this study employed only a surgically induced rat pBOO model. While this model serves as a classic tool for elucidating the pathophysiological mechanisms of pBOO, it simulates only the single causative factor of mechanical obstruction. This contrasts with the complexity of human female urinary tract anatomy and pBOO etiology (e.g., urethral stricture, pelvic organ prolapse, detrusor -urethral sphincter dyssynergia). Third, this study lacks validation using human cells and clinical samples. It did not conduct *in vitro* intervention experiments with human bladder smooth muscle cells or renal tubular epithelial cells, nor did it incorporate tissue specimens or serum samples from clinical pBOO patients for correlation analysis.Additionally, it has been reported that IR-780 can induce mitochondrial ROS by directly inhibiting the functional α-subunit of succinate dehydrogenase (SDH, mitochondrial complex II) and the activity of SDH2 (Wang Z. et al., 2021); however, this was not addressed and requires further verification in subsequent studies. The direct mechanism by which IR-780 ameliorates pBOO-related renal injury has not yet been experimentally verified, representing a key area requiring further investigation in this study.

In summary, This study confirms that IR-780 exhibits mitochondrial targeting properties in bladder smooth muscle cells. By activating the Nrf2/Keap1 pathway, it initiates the cell’s endogenous antioxidant defense system and promotes the expression of antioxidant proteins. This mechanism protects BSMCs and their mitochondria, ultimately improving urinary dysfunction and related complications in rats with pBOO. These findings suggest its potential as a targeted therapy for bladder dysfunction in pBOO. From a clinical translation perspective, determining the optimal administration timing is a critical prerequisite for guiding precise clinical dosing. Subsequent studies will specifically design multi-time-window dosing comparison experiments. By integrating multidimensional indicators such as urodynamic testing, histopathological scoring, and molecular biomarker detection, these studies will systematically evaluate the efficacy differences across varying administration timings. This approach aims to identify the optimal time window for IR-780 intervention in pBOO, providing more precise reference guidelines for its clinical application.

## Data Availability

The original contributions presented in the study are included in the article/Supplementary Material, further inquiries can be directed to the corresponding authors.
