# Supplementary figures and images for: IR-780 improves urination function and complications in rats with partial bladder outlet obstruction by protecting bladder smooth muscle cell mitochondria from oxidative stress
Source: Front Pharmacol. 2026 Feb 27;17:1778496. doi: 10.3389/fphar.2026.1778496 (PMC12982359; doi:10.3389/fphar.2026.1778496)

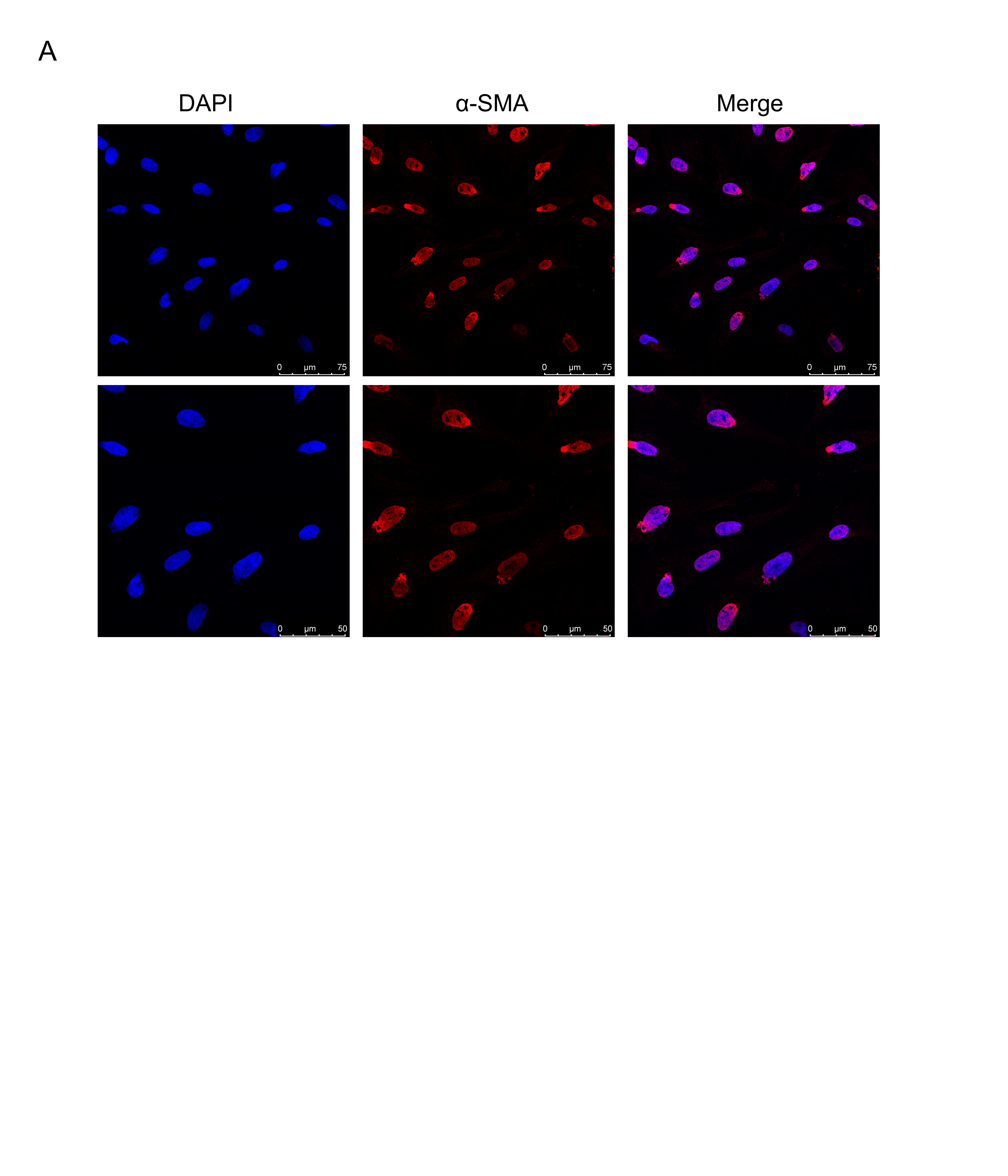

Supplement: Supplementary file 1 [file Image1.tif]
