# Supplementary material for: IR-780 improves urination function and complications in rats with partial bladder outlet obstruction by protecting bladder smooth muscle cell mitochondria from oxidative stress
Source: Front Pharmacol. 2026 Feb 27;17:1778496. doi: 10.3389/fphar.2026.1778496 (PMC12982359; doi:10.3389/fphar.2026.1778496)
Supplement: Supplementary file 2 [file Table1.doc]

Table 1. Primers for quantitative RT-PCR

| **Target** | **Sequence (5'-3')** |
| --- | --- |
| IL-1β | Forward:GTGGAGCTTCCAGGATGAGG  Reverse:CACACACTAGCAGGTCGTCA |
| IL-6 | Forward:CACTTCACAAGTCGGAGGCT  Reverse:TCTGACAGTGCATCATCGCT |
| Tnfa | Forward:CAGCAGATGGGCTGTACCTT  Reverse:AAATGGCAAATCGGCTGACG |
| Hif1α | Forward:AAGTCAGCAACGTGGAAGGT  Reverse:CGGCTGGTTACTGCTGGTAT |
| IL-10 | Forward:GCTCAGCACTGCTATGTTGC  Reverse:TTGTCACCCCGGATGGAATG |
| TGF-β1 | Forward:GACTCTCCACCTGCAAGACC  Reverse:GGACTGGCGAGCCTTAGTTT |
| Col1a1 | Forward:GTACATCAGCCCAAACCCCA  Reverse:CAGGATCGGAACCTTCGCTT |
| Col3 | Forward:ATATGTGTCTGCGACTCGGG  Reverse:GGGCAGTCTAGTGGCTCATC |
| Gapdh | Forward:GACATGCCGCCTGGAGAAAC  Reverse:AGCCCAGGATGCCCTTTAGT |
| Actb | Forward:TGTCACCAACTGGGACGATA  Reverse:GGGGTGTTGAAGGTCTCAAA |

Col1a1,collagen type I-a1;Col3, collagen type III; Hif1a, hypoxiainducible factor-1a; IL, interleukin; Tnfa, tumor necrosis factor-a;Actb, b-actin.
